# Qiang Xin 1 Formula Suppresses Excessive Pro-Inflammatory Cytokine Responses and Microglia Activation to Prevent Cognitive Impairment and Emotional Dysfunctions in Experimental Sepsis

**DOI:** 10.3389/fphar.2020.00579

**Published:** 2020-05-08

**Authors:** Xuerui Wang, Xiaolong Xu, Yuhong Guo, Po Huang, Yanxiang Ha, Rui Zhang, Yunjing Bai, Xuran Cui, Shasha He, Qingquan Liu

**Affiliations:** ^1^ Beijing Hospital of Traditional Chinese Medicine, Capital Medical University, Beijing, China; ^2^ Beijing Institute of Traditional Chinese Medicine, Beijing, China; ^3^ Beijing Key Laboratory of Basic Research with Traditional Chinese Medicine on Infectious Diseases, Beijing, China

**Keywords:** sepsis, cognitive impairment, emotional dysfunctions, traditional Chinese medicine, neuroinflammation, microglia

## Abstract

Sepsis commonly leads to acute and long-term cognitive and affective impairments which are associated with increased mortality in patients. Neuroinflammation characterized by excessive cytokine release and immune cell activation underlies the behavioral changes associated with sepsis. We previously reported that the administration of a traditional Chinese herbal Qiang Xin 1 (QX1) formula improves survival in septic mice. This study was performed to better understand the effects and the mechanisms of QX1 formula treatment on behavioral changes in a preclinical septic model induced by cecal ligation and puncture. Oral administration of QX1 formula significantly improved survival, alleviated overall cognitive impairment and emotional dysfunction as assessed by the Morris water maze, novel object recognition testing, elevated plus maze and open field testing in septic mice. QX1 formula administration dramatically inhibited short and long-term excessive pro-inflammatory cytokine production both peripherally and centrally, and was accompanied by diminished microglial activation in septic mice. Biological processes including synaptic transmission, microglia cell activation, cytokine production, microglia cell polarization, as well as inflammatory responses related to signaling pathways including the MAPK signaling pathway and the NF-κB signaling pathway were altered prominently by QX1 formula treatment in the hippocampus of septic mice. In addition, QX1 formula administration decreased the expression of the M1 phenotype microglia gene markers such as *Cd32*, *Socs3*, and *Cd68*, while up-regulated M2 phenotype marker genes including *Myc*, *Arg-1*, and *Cd206* as revealed by microarray analysis and Real-time PCR. In conclusion, QX1 formula administration attenuates cognitive deficits, emotional dysfunction, and reduces neuroinflammatory responses to improve survival in septic mice. Diminished microglial activation and altered microglial polarization are involved in the neuroprotective mechanism of QX1 formula.

## Introduction

Sepsis, a life-threatening multi-organ dysfunction caused by a dysregulated of host response to infection that is a global challenge due to its high mortality. It is associated with an increased annual incidence and substantial sequelae in patients that survive (Singer et al., 2016). Sepsis affects approximately 100 people per 100,000 annually worldwide (Moss, 2005). Sepsis frequently leads to diffuse brain dysfunction, which is called as sepsis-associated encephalopathy (SAE). Up to 70% of patients with severe sepsis suffer from complications, which clinically are characterized by altered behavior, delirium, coma, and seizures during the acute brain dysfunction stage, and late cognitive and emotional impairment in survivors (Gofton and Young, 2012). Emerging evidence suggests a relationship between long-term cognitive impairment and increased mortality in sepsis survivors (Angus et al., 2001; Iwashyna et al., 2010). Although efforts have been made in the past decades, there is no specific treatment available in routine practice for SAE patients, prompting the need of more specific and effective therapies.

The pathophysiological processes of SAE are highly complex and incompletely understood. A growing amount of evidence has demonstrated that SAE can be triggered by excessive neuroinflammation (Reis et al., 2017), microglial cell activation (Michels et al., 2015), abnormal neurotransmitter release (van Gool et al., 2010), and blood–brain barrier breakdown (Danielski et al., 2018). The excessive and sustained systemic inflammation occurring during sepsis induces the trafficking of inflammatory mediators, including circulating immune cells, chemokines, and cytokines into the brain, leading to activation of neurons and glial cells to evoke pro-inflammatory cytokine responses (Annane et al., 2005). Microglia, the tissue macrophages in the central nerve system (CNS), are the first line of defense to modulate CNS repair and are associated with the initiation and progression of neuroinflammatory responses in SAE (Cunningham, 2013). With sepsis, inflammatory mediators induce rapid changes of microglia from their resting state into an activated state that triggers the production of more inflammatory mediators (Moraes et al., 2015). Activated microglia possesses pro-inflammatory properties, but also contributes to tissue remodeling depending on their polarization into different phenotypes (Michels et al., 2019a). The M1 phenotype, also defined as “classically activated” microglia, is associated with the production of multiple pro-inflammatory cytokines such as tumor necrosis factor-α (TNF-α), interleukin (IL)-1β, and IL-6 (Gordon and Martinez, 2010). These cytokines amplify inflammatory responses and exacerbate cognitive and emotional impairment by activating target cells and accelerating the secretion of other cytokines and chemokine (Mina et al., 2014). The M2 phenotype, also defined as “alternatively activated” microglia, execute an anti-inflammatory and neuroprotective action which promote wound healing and tissue repair in sepsis (Michels et al., 2019b). As multifactorial and complex as the pathology of SAE is, an intervention targeting neuroinflammation, microglial activation and polarization might provide promising therapeutic approaches in preventing cognitive and emotional dysfunction after sepsis.

We have reported previously that a traditional Chinese medicine (TCM) formula, Qiang Xin 1 (QX1) formula treatment reduced mortality in septic mice (Xu et al., 2018). However, little is known about the effects and mechanisms of QX1 formula treatment on cognitive and emotional impairment in sepsis. This study was designed to comprehensively evaluate the effects of QX1 formula administration on behavior with sepsis by conducting a series of experiments focusing on learning and memory, anxiety-like behaviors, and exploratory activities. Based on the cues, we further explored the neuroprotective mechanisms that related to microglia activation and polarization to provide more information about the effects of QX1 formula treatment with sepsis.

## Methods

### Animals

Male C57BL/6 mice (8–10 weeks, 18–22 g) were purchased from Vital River Laboratory Animal Technology Co. Ltd (Beijing China). All the animals were entrained to a controlled temperature (24 ± 0.5°C), 12 h light and 12 h dark cycles (light 08:00–20:00 h; darkness (20:00–08:00 h), and had free access to food and tap water. All animal experimental procedures were approved by the Experimental Ethics Committee of the Institutional Animal Care and Use Committee of Beijing Institute of Traditional Chinese Medicine (application number 2019010216), and all procedures were performed in accordance with the Guideline for the Care and Use of Laboratory Animals from the National Institutes of Health, USA.

### Preparation of Cecal Ligation and Puncture (CLP) Model

The sepsis model was induced by CLP as previously described (Xu et al., 2018). Briefly, mice were anesthetized with pentobarbital sodium (40 mg/kg) by intraperitoneal injection, and a midline incision was made in the lower abdomen. The cecum was ligated using 3.0 silk and punctured with a 5-gauge needle (1 cm from the ileocecal junction). The cecum was gently squeezed to extrude a small amount of feces from the perforation site. Next, the cecum was carefully returned to the peritoneal cavity and the incision was sewed up with 4.0 silk sutures. Mice were injected with 1 ml 37 °C saline after CLP surgery. All animals were returned to their cages had free access to food and water. In the sham operation group, mice were subjected to all the surgical procedures, but the cecum was neither ligated nor perforated.

### Preparation of QX1 Formula

QX1 formula was prepared and decocted as previous described (Xu et al., 2018). Briefly, five Chinese herbal including Shui hong hua zi (*Polygonum orientale L.*) (Voucher number 2019011071), Huang qi [*Astragalus membranaceus (Fisch.) Bge.* var. *mongholicus (Bge.) Hsiao*] (Voucher number 2019011072), Fu ling [*Poria cocos (Schw.) Wolf*] (Voucher number 2019011073), Dan shen (*Salvia miltiorrhiza Bge.*) (Voucher number 2019011074), and Wu wei zi (*Schisandra chinensis (Turcz.) Baill.*] (Voucher number 2019011075) were deposited in Beijing Institute of Traditional Chinese Medicine. Crude drugs of the QX1 formula were soaked and decocted using distilled water for 30 min, and then concentrated to a 1 g/ml final dosage.

### Ultra Performance Liquid Chromatography-Tandem Mass Spectrometry (UPLC–MS/MS) Analysis

A Waters UPLC-MS/MS spectrometer equipped with a HESI-II probe was employed. The details of the setting were as previously described (Xu et al., 2018). The LC-MS system was controlled using Masslynx 4.1. Waters and data were collected and processed with the same software.

### Treatment Protocols

QX1 formula was administrated daily, orally with 0.5 g, 1 g, or 2 g/kg·bw considered as low, medium and high dosage in the manuscript. All sham and CLP mice were treated with the same volume of saline as the vehicle treatment. The flow chart of survival experiments were showed (**Figure 2D**). In the first survival experiment, mice were treated with a low, medium or high dose of QX1 formula beginning at 2 h after CLP surgery. In the second survival experiment, mice were treated with the medium or high dose of QX1 formula beginning at 24 h before CLP surgery. In the third survival experiment, mice were treated with a high dose of the QX1 formula starting at 24 h, 48 h, or 72 h after CLP surgery. For the behavioral and other experiments, mice were randomized divided into sham, CLP, CLP treated with QX1 formula, and CLP treated with dexamethasone (DEX) groups. The flow chart of the behavioral tests were showed (**Figures 3G**, **4I**). Mice were treated with high dose QX1 formula or DEX (sc-204715, Santa Cruz Biotechnology, Germany) beginning at 2 h after CLP surgery. DEX was used as a positive control and mice were treated daily by intraperitoneal injection with 1 mg/kg (Sayed et al., 2018).

### Morris Water Maze (MWM) Test

The MWM test was performed in a circular water tank that was 100 cm in diameter and 40 cm in height. The water temperature was maintained at 22 ± 1°C. The pool was divided into four quadrants (northeast, northwest, southeast, and southwest). A removable hidden platform (5 cm in diameter) was placed in the target quadrant (northeast) at a depth of 0.5 cm below the surface of the water. Each mouse was subjected to three training trials per day for four consecutive days. The training began at day 6 after CLP surgery. Mice were given 90 s to search for the platform. Once a mouse located the submerged platform, it was allowed to remain on it for 10 s. The escape latency, swimming distance and swimming speed were recorded. After each trial, the mice were placed back into their home cages and then given 10 min before the next trial began. On the 5th day, a probe trial was conducted by removing the platform and the mice were permitted to swim freely for 90 s. The time spent in target quadrant was recorded.

### Novel Object Recognition Task (NORT)

The NORT was used to evaluate non-aversive and non-spatial memory. The task was performed 10 days after CLP surgery. Mice underwent a habituation phase, familiarization phase, and discrimination phase for this task. During the habituation phase, mice were placed in a square open field (40×40×40 cm) individually to explore freely for 10 min. No objects were placed in the box during the habituation phase. Twenty-four hours after habituation, the animals were entered into the familiarization phase. In this phase, the mouse individually explored an open field with two identical objects (A1 and A2) that were positioned in two adjacent corners, 10 cm from the walls for 10 min. After 3 h, the mouse was returned to the open field, with one of the familiar objects (A1) replaced by a novel object (A3). For the discrimination phase, each mouse was allowed to explore for 10 min and the time for exploring each object was recorded. Exploration was defined as sniffing, touching the object with the nose and/or forepaws, or facing the object within 2 cm around. The preference index was determined as [time spent in exploring the novel object (A3)/time spent in exploring the two objects (A2 and A3)] × 100%. All the objects and field were cleaned with 75% ethanol between each trial.

### Open Field Task (OFT)

The OFT was carried out in a square open field (40×40×40 cm). The task was performed 10 days after CLP surgery. The field was divided into 25 grids with black lines and the central 9 grids were defined as the central area. The animals were placed on the left rear quadrant and left to explore freely for 5 min (training session). After this, the animals were taken back to their home cage and 24 h later had a similar open field session (test session). The number of times the animal crossed the black lines and demonstrated rearing behavior in both sessions was counted. Time spent in the inner corridor and travel distance was also automatically tracked. The field was cleaned with 75% ethanol between each session.

### Elevated Plus Maze (EPM) Test

The EPM test was used to evaluate anxiety-like behavior. The maze was placed on an aluminum stand 60 cm above the floor. The maze is a cross-shaped maze with two open arms (open areas) and two closed arms (closed areas). The arms were 40 cm in length and 10 cm in width and the two closed arms were enclosed by walls with height of 20 cm, while open arms have no walls. At day 10 after CLP surgery, mice were placed in the central square facing one of the open arms and allowed to explore individually for 5 min. The maze was cleaned with 75% ethanol between each test. Time spent and the numbers of entries in the open and closed arms were calculated.

### Enzyme-Linked Immunosorbent Assay (ELISA)

The mice were anesthetized on the 1st and 10th day after CLP surgery. Then the blood was collected from the apex of heart and the tissues of hippocampus and cortex were collected on ice. The blood was centrifuged at 3000 rpm for 10 min to obtain the serum. The IL-1β, TNF-α, and IL-6 levels in the hippocampus, cortex and serum were analyzed with an IL-1β, TNF-α, and IL-6 Kit II (Boster Biological). Absorbance in each well was measured using a microplate reader (Thermo Fisher, USA) at 450 nm. The results are presented as pg/mg protein in tissue and as pg/ml in serum. Immunofluorescence.

Under anesthesia with pentobarbital sodium (40 mg/kg) by intraperitoneal injection the animals were euthanized, the brains were then removed and fixed in paraformaldehyde at 4°C for more than 24 h. Thereafter, 30% sucrose was used for dehydration. Fixed at an optimal cutting temperature compound at -30°C, brains were cut into 10-μm-thick slices on a cryostat. Brain sections were blocked with goat serum and incubated with primary antibodies Iba-1 (1:1000, Abcam) at 4°C overnight. Subsequently, sections were incubated for 1 h at room temperature with secondary antibodies. Immunofluorescence of Iba1 in the CA1 area of hippocampus was analyzed. The numbers of microglia overlapping with DAPI per μm^2^ and the areas of microglia body per nm^2^ were calculated. All images were captured with a fluorescence microscope (ECLIPSE 90i, Nikon, Japan) and evaluated blindly by a second investigator.

### RNA Extraction and Microarray-Based Transcriptional Profiling

Total RNA was extracted from the hippocampus using a Trizol Reagent (Invitrogen) according to the manufacturer’s instructions. Illumina HiSeq™ 2000 was employed for RNA sequencing. Total RNA treated with DNase I and then enriched by the oligo (dT) magnetic beads was determined. Scanning was performed according to the Illumina HiSeq™ 2000 analysis technical manual. Further bioinformatic analysis, including Kyoto Encyclopedia of Genes and Genomes (KEGG) pathway enrichment analysis and Gene Ontology (GO) enrichment analysis were performed. Values presented are log^2^ RMA signal intensity. According to the KEGG and GO database, we gained functions or pathways of all the target genes. Then, Fisher’s exact test and X^2^ test were used to calculate p values and false discovery rate (FDR) of all the target genes. According to the standard of p < 0.05, significant functions and pathways were filtered.

### Real-Time PCR Analysis

Two micrograms of total mRNA was used for first-strand cDNA synthesis with AMV reverse transcriptase (Promega, Madison, WI). The mRNA levels of genes of interest were analyzed by Real-time PCR, which was performed with 2x SYBR master mix (Takara, Otsu, Shiga, Japan), using a BIORAD iCycler iQ5 (Bio-Rad, Hercules, CA). The sequences accession of the primers are Cd32 (accession # NM_010187), Socs3 (accession # NM_007707), Cd86 (accession # NM_019388), myc (accession # NM_001177352), Arg-1 (accession # CT010173), Cd206 (accession # NM_008625), GAPDH (accession # AY618199). Gene levels were normalized to that of GAPDH. All samples were run in duplicate.

### Data Analysis

Data are expressed as mean ± SD. The statistical software SigmaStat package (SPSS19.0 Inc) was used for data analysis. One-way or two-way ANOVA with or without repeated measures was used, as appropriate, to assess group means, followed by the Scheffe´ multiple-range test for *post hoc* assessment of individual means. A *p* value < 0.05 was considered to be statistically significant. All the data in the manuscript were analyzed by the investigator who was blind to the experimental groups.

## Results

### Identification of Major Components of QX1 Formula

**Figure 1:**
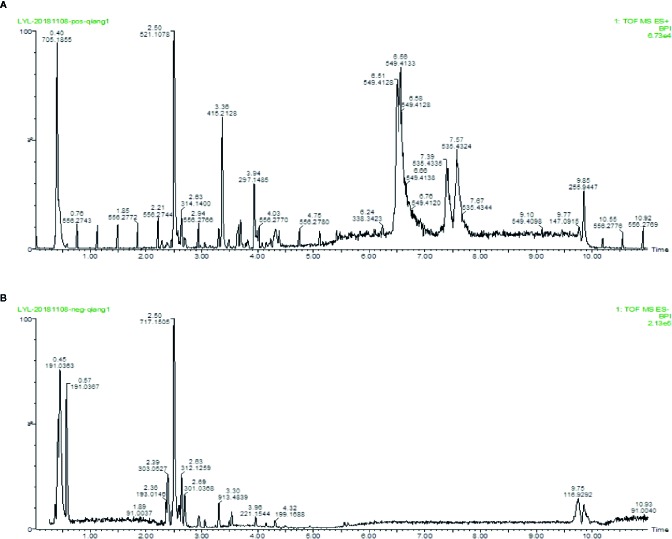
Identification of major components of QX1 formula. QX1 formula sample were examined using UPLC–MS/MS. Base peak intensity chromatogram in the positive **(A)** and negative **(B)** ion modes for QX1 formula were shown as indicated. UPLC–MS/MS, Ultra Performance Liquid Chromatography-Tandem Mass Spectrometry.

To identity the main ingredients, samples of QX1 formula were evaluated by UPLC–MS/MS. The total positive (**Figure 1A**) and negative (**Figure 1B**) ion chromatograms of QX1 formula demonstrated the composition and content of all ingredients. The average contents of eight major component including tanshinone IIA, sodium danshensu, ryptotanshinone, texifolin, quercetin, gallic acid, formononetin, and kaempferoland in three parallel samples are shown in **Table 1**.

**Table 1:** Content of dominating compounds.

Identification	Formula	Average content (μg/ml)
Tanshinone IIA	C_19_H_18_O_3_	105.95
Sodium Danshensu	C_9_H_9_NaO_5_	61.68
Cryptotanshinone	C_19_H_20_O_3_	56.64
Texifolin	C_15_H_12_O_7_	38.04
Quercetin	C_15_H_10_O_7_	29.05
Gallic acid	C_7_H_6_O_5_	22.90
Formononetin	C_16_H_12_O_4_	4.40
Kaempferol	C_15_H_10_O_6_	3.08

### QX1 Formula Improves Survival in Septic Mice

**Figure 2:**
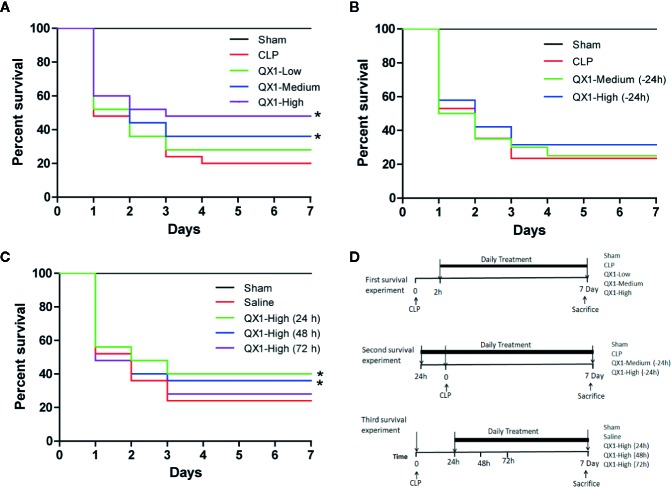
QX1 formula improves survival in septic mice. Kaplan–Meier curves for 7 days demonstrate the survival rate. **(A)** Percent survival of mice orally administrated QX1 formula 2 h after surgery. The dosages indicated as low, medium, and high dose were 0.5 g, 1 g, and 2 g/kg·bw, respectively. (n = 25 per group). **(B)** Percent survival of mice orally administrated QX1 formula 24 h before sepsis onset. The dosages used were medium or high dose with 1 or 2 g/kg·bw respectively. (n = 25 per group). **(C)** Percent survival of mice orally administrated QX1 formula 24 h, 48 h, or 72 h after surgery. The dosages used were high dose with 2 g/kg·bw. (n = 25 per group). **(D)** The flow chart of survival experiments. *p < 0.05 vs. CLP, one-way ANOVA.

To provide insight into the efficacy, dosage and the treatment time window of QX1 formula, CLP, a widely used preclinical severe polymicrobial the sepsis model was induced and the survival rates were calculated. All sham animals survived throughout the 7-day period. After CLP, the medium and high dose of QX1 formula administration significantly improved the survival rate as compared to vehicle (**Figure 2A**). Next, the preventive effects of QX1 formula were evaluated. We found that the medium or high dose of QX1 formula administration 24 h prior to sepsis onset did not result in significant survival alterations (**Figure 2B**). Finally, the high dose of QX1 formula treatment initiated at 24 h or 48 h after CLP surgery significantly improved the survival rate (**Figure 2C**). However, when administered was begun at 72 h after sepsis, the high dose of QX1 formula showed no therapeutic effect (**Figure 2C**). We then chose the high dose of QX1 formula administration as the treatment protocol for the following experiments.

### QX1 Formula Ameliorates Spatial and Non-Spatial Memory Deficits in Septic Mice

**Figure 3:**
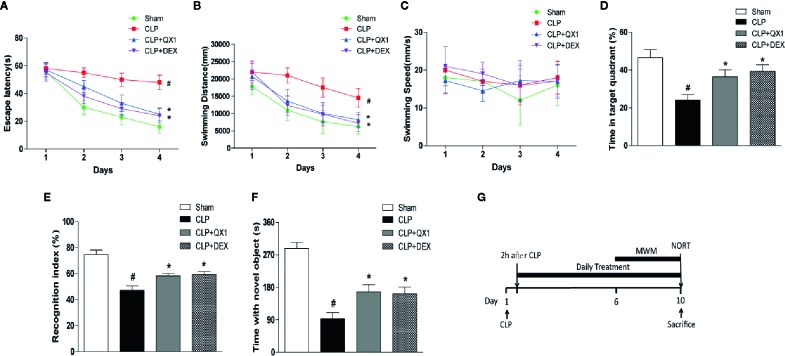
QX1 formula ameliorates cognitive deficits in septic mice. Mice were randomized divided into sham, CLP, CLP treated with QX1 formula, and CLP treated with DEX groups. Mice were treated with QX1 formula (2 g/kg) or DEX (1 mg/kg) beginning at 2 h after CLP surgery. DEX was used as a positive control. In the Morris water maze test, **(A)** Escape latency, **(B)** swimming distance, **(C)** swimming speed, and **(D)** the percentage of time spent in the target quadrant on the fifth day are presented respectively. **(E)** Recognition index, and **(F)** time spend with novel object in the novel object recognition task were presented respectively. Data were shown as mean ± SD (n=8-12 per group). **(G)** The flow chart of Morris water maze test and novel object recognition task. ^#^ *p < 0.05* vs. sham, *p < 0.05 vs. CLP, For Morris water maze test, two-way ANOVA; for novel object recognition task, one-way ANOVA.

To better clarify the effects of QX1 formula on spatial learning and memory impairments in septic mice, escape latency, swimming distance, swimming speed, and the percentage of time spent in the target quadrant were measured by MWM was evaluated. The latency to reach the hidden platform (**Figure 3A**) and swimming distance (**Figure 3B**) of CLP mice were significantly longer than sham. DEX and QX1 formula administration significantly decreased the escape latency and swimming distance compared with CLP vehicle treated mice. The swimming speed showed no differences among all groups (**Figure 3C**), indicating no functional motor impairment in these groups. In the spatial probe trials, total time spent in target quadrant was significantly decreased in CLP mice in comparison with sham. DEX and QX1 formula administration significantly increased the time duration compared with CLP mice (**Figure 3D**).

The effects of QX1 formula on working memory and exploratory behaviors were evaluated by NORT. Regarding working memory, we found that CLP mice exhibited a significantly decreased exploratory preference (**Figure 3E**) and less time with the novel object (**Figure 3F**) compared with the sham group. In contrast, DEX and QX1 formula administration remarkably mitigated sepsis induced recognition memory impairments when compared to CLP mice (**Figures 3E, F**).

### QX1 Formula Ameliorates Emotional Dysfunction in Septic Mice

**Figure 4:**
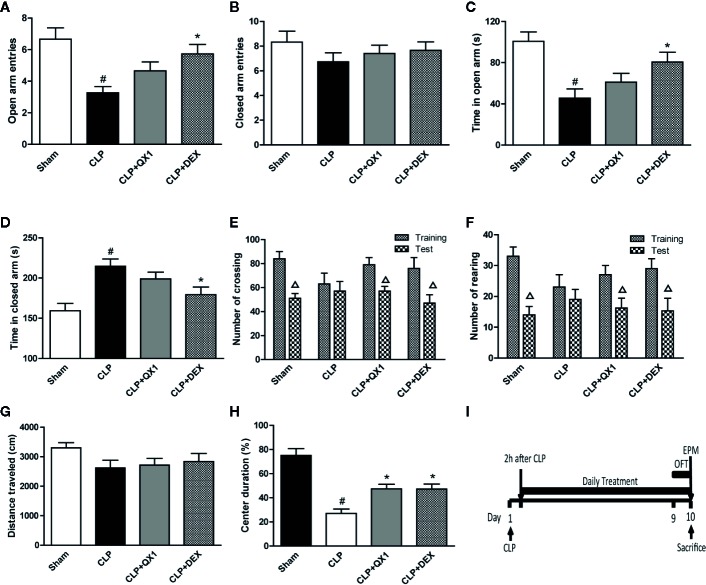
QX1 formula ameliorates emotional dysfunction in septic mice. Mice were randomized divided into sham, CLP, CLP treated with QX1 formula, and CLP treated with DEX groups. Mice were treated with QX1 formula (2 g/kg) or DEX (1 mg/kg) beginning at 2 h after CLP surgery. DEX was used as a positive control. In the elevated plus maze, **(A)** open arm entries, **(B)** closed arm entries, **(C)** time spent in the open arms, and **(D)** time spent in the closed arms were evaluated respectively. In the open field task, **(E)** the number of crossings and **(F)** rearings, **(G)** total distance traveled, and **(H)** percentage of center duration in the 5 min test were presented respectively. **(I)** the flow chart of open field task and elevated plus maze test. Data were shown as mean ± SD (n=8-12 per group). ^#^ *p < 0.05* vs. sham, *p < 0.05 vs. CLP, ^△^ *p < 0.05* vs. training, one-way ANOVA. CLP, Cecal Ligation and Puncture; QX1, Qiang Xin 1; DEX, dexamethasone.

To analyze the effects of QX1 formula on anxiety-like behaviors, EPM and OFT were carried out. In the EPM testing, we found that CLP mice made significantly fewer entries into the open arms (**Figure 4A**), spent less time in the open arms (**Figure 4C**), and spent more time in the closed arms (**Figure 4D**) than sham mice. DEX administration increased the number of entries and the time in the open arms, and decreased the time in the closed arms as compared to CLP mice. QX1 formula treatment showed a trend towards increasing the number of entries, time in the open arms, and decreasing the time in the closed arms, but that was not statistically different as compared to CLP mice. The number of entries into the closed arms showed no difference among all groups (**Figure 4B**).

In the OFT testing, the number of crossings and rearings were recorded to evaluate motor and exploratory activity in animals. In the habituation section, the number of crossings and rearings showed no difference among all groups. Significant reductions emerged in both crossings (**Figure 4E**) and rearings (**Figure 4F**) in the test section that compared the training section in sham, DEX, and QX1 formula treated mice. However, the number of crossings and rearings in CLP mice showed no difference on this test. The total distance traveled was also recorded to evaluate general locomotor behavior. No differences were found among all groups (**Figure 4G**). Central duration in the open field evaluates anxiety-like behaviors. We found that CLP mice spent less time in the central area than that of the sham group, and DEX and QX1 formula administration significantly increased the central duration time in comparison with CLP mice treated with vehicle (**Figure 4H**).

### QX1 Formula Ameliorates Peripheral and Central Inflammatory Responses in Septic Mice

**Figure 5:**
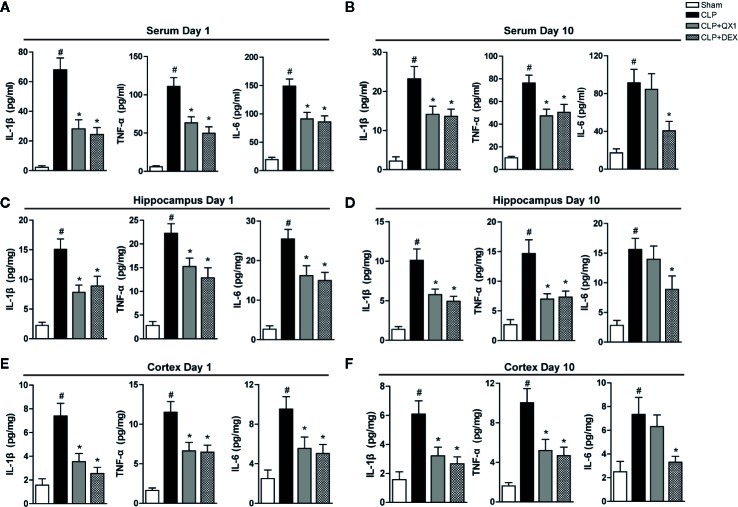
QX1 formula ameliorates peripheral and central inflammatory responses in septic mice. Mice were randomized divided into sham, CLP, CLP treated with QX1 formula, and CLP treated with DEX groups. Mice were treated with QX1 formula (2 g/kg) or DEX (1 mg/kg) beginning at 2 h after CLP surgery. DEX was used as a positive control. Levels of inflammatory factors were determined by ELISA. Levels of TNF-α, IL-1β, and IL-6 at day 1 **(A)** and day 10 **(B)** in serum of septic mice. Levels of TNF-α, IL-1β, and IL-6 at day 1 **(C)** and day 10 **(D)** in the hippocampus of septic mice. Levels of TNF-α, IL-1β, and IL-6 at day 1 **(E)** and day 10 **(F)** in the frontal cortex of septic mice. Data were shown as mean ± SD (on day 1, n=12-15 per group; on day 10, n=15 per group). ^#^ *p < 0.05* vs. sham, *p < 0.05 vs. CLP, one-way ANOVA. CLP, Cecal Ligation and Puncture; QX1, Qiang Xin 1; DEX, dexamethasone.

To determine the effect of QX1 formula on regulating inflammatory responses, the levels of cytokines were measured both peripherally and centrally in septic mice. At day 1 after surgery, the levels of pro-inflammatory mediators, including IL-1β, TNF-α, and IL-6 were up-regulated sharply in serum, the hippocampus and the frontal cortex of CLP mice compared with sham. DEX and QX1 formula administration significantly mitigated the levels of IL-1β, TNF-α, and IL-6 in serum (**Figure 5A**), the hippocampus (**Figure 5C**) and the frontal cortex (**Figure 5E**) compared with CLP vehicle treated mice. At day 10 after surgery, the levels of IL-1β, TNF-α, and IL-6 in serum (**Figure 5B**), the hippocampus (**Figure 5D**), and the frontal cortex (**Figure 5F**) were up-regulated in CLP mice compared with sham. DEX treatment significantly reduced the levels of IL-1β, TNF-α, and IL-6 in serum, the hippocampus and the frontal cortex of septic mice. Meanwhile, QX1 formula administration down-regulated the levels of IL-1β and TNF-α in serum, the hippocampus and the frontal cortex, but showed no effects on the levels of IL-6.

### QX1 Formula Reduces Microglia Activation in Septic Mice

**Figure 6:**
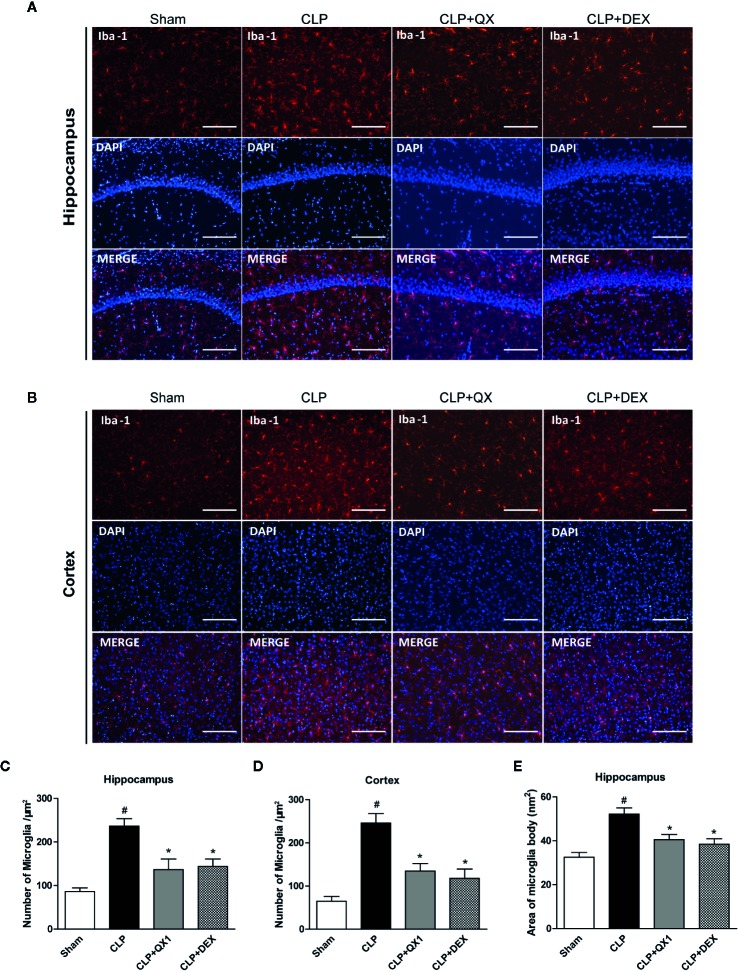
QX1 formula reduces microglia activation in septic mice. Representative Iba-1 and DAPI immunofluorescence staining in **(A)** the CA1 area of the hippocampus and **(B)** the frontal cortex in mice treated with QX1 formula or DEX beginning at 2 h after CLP surgery. Scale bar = 100 μm. **(C, D)** Statistical analysis of Iba-1 positive cell counts in the hippocampus and the frontal cortex. **(E)** Statistical analysis of Iba-1 positive cell body area in the hippocampus (n=10 per group, three slices per animal). Data were shown as mean ± SD. ^#^ *p < 0.05* vs. sham, *p < 0.05 vs. CLP, one-way ANOVA. QX1, Qiang Xin 1; CLP, Cecal Ligation and Puncture.

To determine the effect of QX1 formula on the activation of microglia, Iba-1 was stained in the hippocampus and the frontal cortex at 10 days after sepsis onset. Large numbers of Iba-1 positive cells were observed in the hippocampus (**Figure 6A**) and the frontal cortex (**Figure 6B**) of CLP mice, whereas very few such cells were visible in the sham. It should be emphasized that DEX and QX1 formula administration decreased the numbers of Iba-1 positive cells in the hippocampus (**Figure 6C**) and the frontal cortex (**Figure 6D**) relative to CLP mice. DEX and QX1 formula administration also significantly decreased the areas of microglia body in the hippocampus (**Figure 6E**).

### Characterization of Molecular Events Elicited by QX1 Formula Based on Microarray Genomic Detection and Bioinformatics Analysis

**Figure 7:**
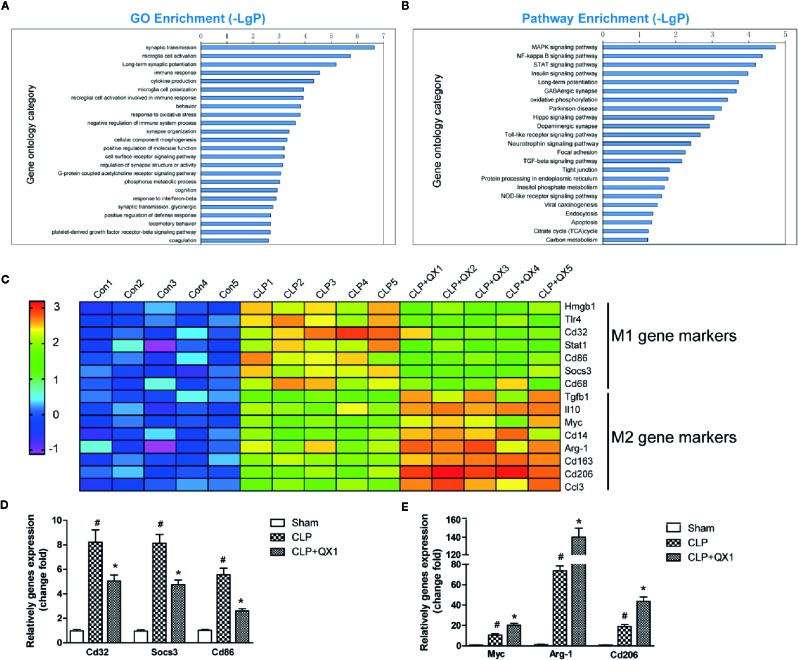
QX1 formula promotes microglia polarization in septic mice. Transcriptional profiling and bioinformatics analysis of the hippocampus in septic mice at day 10 after surgery (n=5 per group). **(A)** GO analysis and **(B)** KEGG pathway enrichment analysis of differential genes. -LgP is the logarithm of *p* value. **(C)** Expression of microglia M1 and M2 polarization related genes in log_2_ value in transcriptional profiling. Realtime-PCR validation of **(D)** M1 and **(E)** M2 microglia polarization related genes identiﬁed from the microarray analysis upon CLP and CLP+QX1 formula treatment (n=12 per group). Data were shown as mean ± SD. ^#^ *p < 0.05* vs. sham, *p < 0.05 vs. CLP, one-way ANOVA. GO, Gene Ontology; KEGG, Kyoto Encyclopedia of Genes and Genomes; CLP, Cecal Ligation and Puncture; QX1, Qiang Xin 1.

To further explore the mechanism involved in sepsis induced inflammation in the brain, microarray analysis were performed in the hippocampus of CLP mice at day 10 after surgery. ANOVA corrected changed genes [p-value and the false discovery rate (FDR) less than 0.05] between these groups were determined. Bioinformatics analysis includes GO and KEGG pathway analysis. The top 8 altered biological processes by QX1 formula treatment were synaptic transmission, microglia cell activation, long-term synaptic potentiation, immune response, cytokine production, microglia cell polarization, microglial cell activation involved in immune response, and behavior (**Figure 7A**). KEGG pathway analysis showed that genes encoding vital regulators including MAPK signaling pathway, NF-κB signaling pathway, STAT signaling pathway etc. were significantly altered after QX1 formula treatment (**Figure 7B**).

### QX1 Formula Promotes Microglia Polarization Toward the M2 Phenotype in Septic Mice

As was indicated by GO analysis, microglial cell activation and polarization might play a pivotal role in QX1 formula elicited neuroprotective effects. We further analyzed gene profiles related to M1/M2 phenotype polarization based on microarray. Genes related to M1 phenotype polarization including *Hmgb1*, *Tlr4*, *Cd32*, *Stat1*, *Cd86*, *Socs3*, *Cd68*, and genes related to M2 phenotype polarization including *Tgfb1*, *Il10*, *Myc*, *Cd14*, *Arg-1*, *Cd163*, *Cd206*, *Ccl3* were up-regulated significantly in CLP hippocampus as compared to sham. A particularly notable observation was that QX1 formula treatment significantly down-regulated these M1 phenotype related genes, while up-regulating M2 phenotype related genes as compared to vehicle treated CLP mice (**Figure 7C**). Realtime PCR further confirmed the same variation trend of M1 genes including *Cd32*, *Socs3,* and *Cd68* (**Figure 7D**), and M2 genes including *Myc*, *Arg-1,* and *Cd206* (**Figure 7E**) in sham, CLP, and QX1 formula treatment groups.

## Discussion

Brain dysfunction with sepsis contributes to its high mortality, and is associated with the mechanisms of the sepsis induced neuroinflammatory responses. Here, we showed for the first time that oral administration of a QX1 formula attenuates cognitive deflects, and emotional dysfunction as well as suppressing peripheral and central cytokine production. It also improved survival in a CLP induced sepsis mouse model. The mechanisms of QX1 formula regulation of microglial activation and microglial polarization were also identified.

Sepsis is a highly lethal clinical syndrome without an effective treatment. We previously reported that the administration of a TCM herbal, QX1 formula, significantly reduced mortality in septic mice (Xu et al., 2018).This herbal formulation has been clinically applied for more than 30 years, and was designed and evolved from studies by Qingquan Liu, the leading drafter of the TCM clinical practice guideline for sepsis patients (Zhao et al., 2019). A large, high-quality randomized controlled trial for the efficacy evaluation of QX1 formula in sepsis patients is still lacking. The ingredients of the QX1 formula were reported to be stable and its efficacy reproducible (Xu et al., 2018). The effective components of the formulation are multifactorial. However, the main components of the QX1 formula that were identified in this study included: tanshinone IIA and cryptotanshinone, has been reported to elicit protective effects in septic animals related to anti-inflammatory and anti-oxidative stress mechanisms (Gao et al., 2018). Tanshinone IIA has been reported to have protective effects in sepsis and other diseases associated with cognitive deficits, related to suppressing microglial activation and neuronal apoptosis in the hippocampus (Chen et al., 2018; Xiong et al., 2019).

The substantial mortality of sepsis is a major challenge for physicians worldwide. Sepsis is a major killer of patients in the ICU and has an approximately 40% mortality rate within 1 year after hospital discharge (Vincent et al., 2009) and 80% after 5 years (Iwashyna et al., 2010). In our previous reports, we found that the medium dose (1 g/kg·bw) of QX1 formula increased the survival of septic mice from 22 to 40% at day 7. Here, we found that high dose (2 g/kg·bw) of QX1 formula had a modestly better effect improving the survival rate up to 50%. However, pretreatment with a therapeutic dose of QX1 formula 24 h before sepsis onset failed to show preventive effect on mortality. QX1 formula administration 24 h to 48 h after sepsis onset also reduced mortality, but administration 72 h after sepsis onset did not reduce mortality rate. These results demonstrated that there is a time window for the beneficial administration of QX1 formula, suggesting that QX1 formula should be administrated as early as possible in clinical practice for sepsis patients.

Clinical manifestations of cognitive and emotional impairment are frequently noted during sepsis from the initial phase, during the recovery phase, and after hospital discharge (Prescott and Costa, 2018). Brain structures including the neuroendocrine system, hippocampus, limbic system, frontal cortex, and brainstem of sepsis patients have been found to be damaged (Annane and Sharshar, 2015). These brain lesions can be associated with psychological disorders (anxiety, depression), long-term cognitive impairment (memory and executive function), and death (Wintermann et al., 2015; Calsavara et al., 2018). Studies have reported that patients with sepsis were three times more likely to develop cognitive impairment than patients without sepsis (Iwashyna et al., 2010). Cognitive impairment was found in 25% of sepsis patients at 1 year after hospital discharge (Needham et al., 2013), and in about 70% at 1 year after ICU discharge (Jackson et al., 2010). Consistent with our results, animal studies using a CLP model reported cognitive and emotional dysfunction at day 10 after surgery (Petronilho et al., 2012; Manfredini et al., 2019). A series of behavioral tests were carried out in our study, and it was noteworthy that the QX1 formula significantly reduced overall hippocampus-regulated learning and memory impairment detected by MWM and NORT, frontal cortex-regulated anxiety-like behaviors detected by OPT and EPM, and increased exploratory activity revealed by NORT and OFT, comprehensively demonstrated the neuroprotective effects of QX1 formula on behavior changes.

An excessive, sustained systemic inflammation is the hallmark of sepsis which contributes to the activation of pro-inflammatory cytokine responses in the CNS (Rosas-Ballina et al., 2015). Previous findings confirmed that the level of TNF-α, IL-1β, and IL-6 are elevated within 6 h in sepsis brain tissue and continually increased for 10 to 30 days (Biff et al., 2013; Jeremias et al., 2016). It is important to note that the increased pro-inflammatory cytokine levels in the brain parenchyma showed a high correlation with memory impairment and the death rate in animal models of sepsis (Moraes et al., 2015; Xu et al., 2019). Similarly, we found an increased TNF-α, IL-1β, and IL-6 levels in the serum, the hippocampus, and the frontal cortex at day 1 and day 10 after sepsis onset. We previously found that the QX1 formula decreased the secretion of IL-1β and TNF-α in the serum of septic mice at 24 h and 48 h after CLP surgery. In addition, we found that QX1 formula treatment decreased TNF-α and IL-1β levels in the serum, the hippocampus, and the frontal cortex of septic mice at day 1 and day 10 after CLP. It also reduced IL-6 secretion in serum, the hippocampus, and the cortex at day 1 after CLP, while no statistically significant reduction was seen at day 10. These results demonstrated the short and long-term effects of the QX1 formula on reducing cytokines release both peripherally and centrally in septic mice.

Microglia, a major type of immune cell in CNS, are important for the initiation and progression of neuroinflammation. Several autopsy studies have demonstrated higher densities of microglia in different brain regions of sepsis patients (Lemstra et al., 2007; Zrzavy et al., 2019). The activated microglia marker, CD68, was reported to be significantly increased in sepsis patients, while the microglia morphological heterogeneity marker irrespective to activation, MHC-II, was not altered (Westhoff et al., 2019). Different from resting microglia, activated microglia are associated with repair, cytotoxicity, and the immune response under pathological conditions (Ransohoff and Cardona, 2010). Interventions targeting the activation of microglia showed powerful effects on reducing oxidative stress, inflammatory responses, and improving long-term cognitive behavior of septic mice (Henry et al., 2008; Michels et al., 2015). Here, we found the numbers of Iba-1, a marker of microglia, were remarkably increased in the hippocampus and the frontal cortex of CLP mice. Consistently, the body areas of microglia cell that represent the morphological alterations of activated microglia, was increased in septic mice. QX1 formula administration showed a significant effect on reducing the activation of microglia in CNS. The phenotypes of activated microglia are classically differentiated into M1 and M2 polarization. M1 microglia usually become predominant in the early phase of infection to control pathogen spread (Benoit et al., 2008), and a shift to the M2 microglial phenotypes after control of the infection to potentially to limit the recruitment of immune cells and reduce tissue damage (Kumar et al., 2016; Michels et al., 2017; Liu et al., 2018). We demonstrated in this study that QX1 formula administration promoted the transfer of microglia polarization from an inflammatory M1 phenotype to a protective M2 phenotype. Assessment of gene expression profiles in a genomics analysis further indicated the involvement of biological processes including microglia cell activation, immune response, cytokine production, microglia cell polarization in the mechanisms of QX1 formula treatment. Vital inflammatory responses related pathways including MAPK signaling pathway, NF-κB signaling pathway, and STAT signaling pathway were altered significantly by QX1 formula treatment. The specific components and the potential targets of QX1 formula administration on microglia activation and polarization, and the crucial signaling pathways involved in the neuroprotective mechanisms of QX1 formula administration need to be further evaluated in future studies.

## Data Availability Statement

The raw data supporting the conclusions of this manuscript will be made available by the authors, without undue reservation, to any qualified researcher.

## Ethics Statement

The animal study was reviewed and approved by The Experimental Ethics Committee of the Institutional Animal Care and Use Committee of Beijing Institute of Traditional Chinese Medicine (application number 2019010216).

## Author Contributions

XW and QL conceived, designed the study, and wrote the manuscript, which as approved by all the authors. XX and YG conducted the behavior tests. PH, YH, and RZ carried out the CLP surgery, drug treatment, and the collection of samples. YB, and SH carried out the immunofluorescence and Elisa evaluation. XC performed data analysis.

## Funding

This work was supported by National Natural Science Foundation of China (No. 81673934, No. 81973608), National Natural Science Foundation of Beijing (No. 7192083), and National Major Science and Technological Project of China (No. 2017XZ10305501).

## Conflict of Interest

The authors declare that the research was conducted in the absence of any commercial or financial relationships that could be construed as a potential conflict of interest.
